# A Qualitative Study to Explore the Influence of Condition Prioritisation in People With Coexisting Diabetes and Hypertension on Medication Adherence

**DOI:** 10.1111/hex.70682

**Published:** 2026-05-04

**Authors:** Pauline Tendai Maniki, Betty Bouad Chaar, Parisa Aslani

**Affiliations:** ^1^ Faculty of Medicine and Health The University of Sydney School of Pharmacy, The University of Sydney Sydney Australia

**Keywords:** chronic conditions, condition management, multimorbidity, nonadherence

## Abstract

**Background:**

Managing multiple chronic conditions often requires people to make treatment decisions, particularly when faced with competing demands. This usually leads to condition prioritisation, where one condition is prioritised over the other. Considering that diabetes and hypertension are closely linked, prioritising medication for one condition over the other can have serious health implications. This study aimed to explore condition prioritisation in people with coexisting diabetes and hypertension, and its impact on medication adherence.

**Methods:**

A qualitative study was conducted with adults on medications to manage coexisting diabetes and hypertension, residing in Australia. Thirty participants were asked to indicate the condition they considered more important to manage and discuss their prioritisation. Thematic analysis was used to identify key factors influencing condition prioritisation. The Adherence to Refills and Medication Scale questionnaire was used to assess medication adherence for each condition.

**Results:**

Medication adherence scores varied in most cases, with diabetes and hypertension scores ranging from 12 to 21 and 12 to 26, respectively. Participants who prioritised one condition over the other demonstrated better medication adherence for the condition they perceived as more important. The key themes influencing disease prioritisation emerged primarily as patient‐related and condition‐related factors. Most participants prioritised diabetes due to its immediate perceived risks, fear of complications and previous experience with the condition.

**Conclusion:**

Participants' perceptions of a condition and observed effects of the condition influenced condition prioritisation. This in turn influenced medication adherence, as participants were more vigilant in managing the condition they prioritised. These findings emphasise the need for tailored interventions that address the challenges of managing multiple conditions and medications.

**Patient or Public Contribution:**

People living with diabetes and hypertension took part as study participants but were not involved in the design, analysis, or dissemination stages of this research. A lay summary of the results will be shared via email with participants who expressed interest in receiving the findings of the study.

## 1 Introduction

Globally, the prevalence of co‐existing conditions is a public health concern [1], especially, as the coexistence of chronic conditions is more prevalent among older adults [2]. It has been estimated that two out of every five individuals with diabetes also have hypertension, thereby presenting a challenge to healthcare systems globally [3, 4]. This duo increases the risk of mortality by 7.2 times, which is primarily attributed to the increased risk of cardiovascular complications [5, 6]. In addition to increasing risks, the coexistence of hypertension and diabetes amplifies the burden by complicating clinical management [7].

Managing both conditions simultaneously requires an active, hands‐on approach from both patients and healthcare providers [8]. Taking this hands‐on approach requires patients to understand both conditions and how they affect each other [8, 9]. For example, uncontrolled diabetes damages blood vessels, making them stiff and less elastic, which increases blood pressure, while uncontrolled hypertension damages insulin‐producing cells in the pancreas, leading to poor insulin secretion and worsening blood sugar control [7, 8, 9]. Moreover, poor control of either condition accelerates kidney damage, and as kidney function deteriorates, managing both hypertension and diabetes becomes more challenging since the kidneys play a crucial role in filtering waste and regulating blood pressure [7, 9].

While it is important to manage both conditions together, patients often struggle with conflicting demands related to treatment adherence, lifestyle changes, and healthcare involvement [10]. This often leads to ‘condition prioritisation’, where one condition's treatment may be prioritised over the other, making it more difficult to manage both conditions effectively [10, 11]. According to Moore‐Bouchard et al. (2024), patients tend to prioritise the condition they regard as more dominant, and this is determined by factors such as symptom intensity, condition severity, and impact on daily life [12]. For instance, a systematic review that looked at the priorities of patients with multimorbidity regarding treatment and health outcomes, reported that condition prioritisation by patients was driven by previous personal and emotional experiences with the condition [13]. However, evidence revealing the underlying reasons behind condition prioritisation remains limited.

Considering that diabetes and hypertension are closely linked, prioritising one condition over the other can have serious implications for long‐term patient outcomes [14]. Focusing more on one condition may lead to inadequate management of the other, disrupting day‐to‐day self‐care activities such as medication adherence, lifestyle modifications, and regular monitoring [13, 15].

To the best of our knowledge, no study has explored factors influencing condition prioritisation in people with coexisting diabetes and hypertension. Therefore, this study aimed to explore condition prioritisation in people with coexisting diabetes and hypertension, and its influence on medication adherence using a descriptive qualitative approach.

## 2 Methods

### 2.1 Study Design

A qualitative study using semi‐structured interviews was conducted to explore condition prioritisation in people with coexisting diabetes and hypertension. Interviews are frequently used in health services research as they allow in‐depth exploration of a specific phenomenon [16]. Ethics approval was obtained from the institutional human research ethics committee (Project number: 2023/584). The consolidated criteria for reporting qualitative research (COREQ) (Additional material 1) was used to provide transparency in data reporting to improve rigour, comprehensiveness, and trustworthiness of the study [17].

An interview guide containing open‐ended probing questions was developed using the Health Belief Model, a theoretical framework useful in understanding health‐related behaviours, expert input, and review of existing literature [18, 19, 20, 21, 22]. Open‐ended questions allow participants to express their opinions more accurately and freely, avoiding the limitations of closed‐ended responses, which may not fully capture their perspectives, enhancing data richness [23, 24, 25]. The interview guide was reviewed for clarity and relevancy by two experienced qualitative researchers (Additional material 2). The interview guide was piloted with experts in the field, who reviewed the questions for clarity and alignment with the study aims, and provided feedback that informed minor refinements.

### 2.2 Participant Recruitment and Data Collection

Participants were recruited through a market research company using their existing panel database. The company contacted individuals via email or telephone, based on pre‐specified eligibility criteria provided by the research team. Recruitment continued until data saturation was reached. They were given a participant information sheet with details about the study, confidentiality, the voluntary nature of the study, and anonymity in the reporting of study findings.

Participants were included if they were:

- 18 years or above.
- Diagnosed with both diabetes and hypertension and on medications for both conditions.
- Residing in Australia at the time of the study.
- Not pregnant.
- Not residing in residential and aged care facilities.

Interviews were conducted between February and May 2024 via Zoom [26]. Verbal consent was given at the beginning of each interview. The mean interview duration was 42 min, with interviews ranging from 30 to 60 min. All interviews were conducted in English by one researcher (PTM) who received prior training in qualitative research. Additionally, a qualitative and medication adherence expert (PA) took part in some of the initial interviews to support interview conduct and ensure quality by refining the questioning techniques. The involvement was meant to enhance methodological rigour and consistency and was not perceived to influence participant responses.

Topics explored in the interview included participants' perception of having diabetes and hypertension, barriers to and facilitators of medication adherence, and participants' perceptions of managing both conditions. As part of the data collection process, participants were asked to indicate which condition they considered more important to manage, with additional perspectives emerging from spontaneous interactions during the interviews. In this study, participants understood condition prioritisation in the context of coexisting diabetes and hypertension as weighing the relative importance of managing each condition when making medication‐related decisions particularly in relation to achieving and maintaining good health outcomes.

Although the interviews explored different perspectives of managing coexisting diabetes and hypertension, this article focuses on condition prioritisation and its impact on medication adherence. Barriers and facilitators of medication adherence and other related findings will be presented in a separate publication.

Demographic information and medical history relevant to the study were also collected. Field notes were recorded during and immediately after each interview to capture contextual information. These notes were used to inform the interpretation of the data. The Adherence to Refills and Medication Scale‐12 (ARMS‐12) questionnaire was used to assess medication adherence for the two conditions independently at the end of each interview [27]. The ARMS‐12 questionnaire was used as it is a valid and reliable measure of both medication‐taking and refill behaviours, especially among people with chronic conditions [27]. Interviews continued until no new themes were generated, after which four additional interviews were conducted to confirm data saturation.

### 2.3 Data Analysis

Interviews were audio‐recorded and transcribed verbatim. Data analysis was conducted using Braun and Clarke's six‐phase thematic analysis approach: familiarisation, coding, theme development, theme review, defining themes and report writing [28, 29]. NVivo software (version 14) was used for data analysis to enable the management of large volumes of qualitative data systematically, ensuring a rigorous and transparent analytical process [30].

The first two interviews were coded by two independent coders to reduce bias and to improve the reliability of the analysis. Coding focused on the reasoning participants used when comparing diabetes and hypertension in medication‐related decision‐making, particularly how participants considered managing their conditions and avoiding perceived harms and consequences. Representative quotes were selected through a consensus meeting with all research team members. Each quote was carefully reviewed and agreed upon by the team to ensure consistency, validity, and alignment with the study's objectives (Additional illustrative quotes have been included in additional material 3). Medication adherence was assessed using the 12‐item ARMS scale with responses recorded on a 4‐point Likert scale, 1 = None of the time, 2 = Some of the time, 3 = Most of the time and 4 = All of the time. The ARMS‐12 tool adherence score ranges from 12 (perfect adherence) to 48 (poorest adherence), with higher scores indicating lower adherence.

To increase the validity of the findings, we used methodological triangulation, by using both qualitative and quantitative approaches, to determine if participants were adherent to their medication. We compared participants' qualitative accounts of medication taking and adherence with their corresponding quantitative adherence scores for each condition. Additionally, the quantitative adherence scores were compared with participants' reports of condition prioritisation for medication taking and adherence. In this way we were able to determine if reported condition prioritisation was reflected in the adherence scores between the two conditions.

## 3 Results

### 3.1 Demographic Characteristics

A total of thirty participants were interviewed (Table 1). More than half were female (60%). The study sample was made up of a diverse population, with participants representing over 10 distinct ethnic groups. Table 1 summarises the demographic characteristics of the sample as reported by participants.

**Table 1:** Demographic characteristics.

Demographic characteristic	Number of participants (*n*)	Relative frequency (%)
**Gender**		
Male	12	40
Female	18	60
**Age (years)**		
30–39	1	3
40–49	5	17
50–59	10	33
60–69	10	33
70–79	3	10
80–89	1	3
**Self‐reported ethnic identity**		
Australian	8	27
Anglo‐Saxon	4	13
Aboriginal and Torres Strait Islander	4	13
Anglo‐Celtic	1	3
Asian	3	10
South African	1	3
Egyptian	1	3
Italian	1	3
Irish	2	7
European	2	7
Fijian	1	3
Middle eastern	1	3
Latino	1	3
**Highest Education Qualification**		
High school	6	20
Certificate^a^	4	13
Diploma^a^	7	23
Bachelor's degree	10	33
Masters degree	3	10
**Years with diabetes**		
≤ 5 years	8	27
6–10 years	8	27
11–15 years	7	23
> 15 years	7	23
**Years with hypertension**		
≤ 5 years	9	30
6–10 years	10	33
11–15 years	3	10
> 15 years	5	17
Unspecified	3	10
**Years with both diabetes and hypertension**		
≤ 5 years	13	43
6–10 years	8	27
11–15 years	3	10
> 15 years	3	10
Unspecified	3	10
**Residential location**		
Metropolitan	28	93
Rural	2	7

^a^ Diplomas offer deeper knowledge over 1–2 years, while certificates focus on specific skills and are often equal to 6–12 months of degree study and they are both post‐school qualifications.

### 3.2 Self‐Reported Condition Prioritisation

Nearly half of the participants (47%) reported that they treated both conditions the same when asked the direct question on condition prioritisation. Some participants described one condition as more important to manage during their narratives but named a different condition or claimed to view the conditions as equal when directly asked which condition they considered more important. Table 2 presents the results of self‐reported condition prioritisation.

**Table 2:** Self‐reported condition prioritisation.

Condition prioritised	Number of participants (*n* = 30)	Relative frequency (%)
Diabetes	10	33
Hypertension	4	13
Equal	14	47
Not specified	2	7

### 3.3 Condition Prioritisation and Medication Adherence Patterns

Individual adherence scores between diabetes and hypertension differed in most cases, indicating that participants did not always manage both conditions in the same way. Out of the 30 participants, 12 (40%) had equal adherence scores for both conditions, 6 (20%) adhered more to their hypertension medication and 12 (40%) adhered more to diabetes medication. Participants reported a mean diabetes adherence score of 15.47 (median 15.0) compared to a mean hypertension adherence score of 16.27 (median 16.0), indicating marginally higher adherence to diabetes medication. Additionally, the most common adherence score (mode) was 13 for diabetes and 15 for hypertension, further suggesting that participants generally reported higher adherence to diabetes medication.

While some participants reported that both conditions were of equal importance, discrepancies in adherence scores were observed. However, a general trend emerged in many cases, showing that participants who regarded the management of one condition as more important showed better adherence to medication for that condition than the other. Table 3 summarises the condition prioritisation and medication adherence patterns. A table detailing individual adherence scores and self‐reported condition prioritisation has been added as Additional Material 4.

**Table 3:** Condition prioritisation and adherence patterns.

Self‐reported prioritised condition	Number (relative frequency %) of participants with equal adherence scores	Number (relative frequency %) of participants with lower diabetes adherence score	Number (relative frequency %) of participants with lower hypertension adherence score
Diabetes	2 (20)	5 (50)	3 (30)
Hypertension	1 (25)	2 (50)	1 (25)
Equal	7 (50)	5 (36)	2 (14)
Not specified	2 (100)	0	0

*Note:* Lower adherence scores indicate better adherence to medication for that condition.

### 3.4 Factors Influencing Condition Prioritisation in People With Coexisting Diabetes and Hypertension

**Figure 1:**
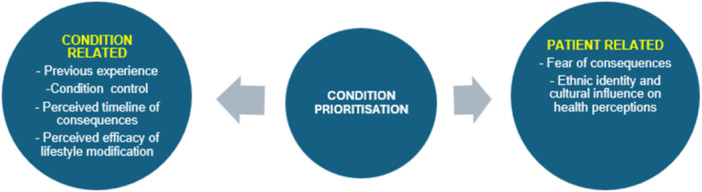
Study themes and subthemes.

Two main themes emerged in exploring condition prioritisation in people with coexisting diabetes and hypertension, namely patient‐related and condition‐related. Sub‐themes identified include ‘Fear of consequences which focused on the emotional response to the nature and severity of the consequences, and ‘Perceived timeline of consequences,’ which focused on participants’ cognitive assessment of when those outcomes occur (e.g., immediate *vs.* delayed). The main themes and sub‐themes identified in this study are summarised in Figure 1.

### 3.5 Patient‐Related Factors

#### 3.5.1 Fear of Consequences

Participants described how fear of the consequences of not treating the condition influenced the condition they prioritised. In most interviews, participants pointed out that they regarded diabetes as the most important condition due to fear of consequences associated with poor diabetes control. As some participants explained:

Diabetes is a lot more important to manage and take care of because of the risks of you know, gangrene and amputation. Blood sugar levels are dangerous to play around with. So, it can be deadly, you know, more so than high blood pressure.

– B29 (B29 refers to participant 29; ‘B’ was used for all participants as part of anonymisation.)

I think the blood pressure is a little bit more important than diabetes. It's only because diabetes cannot give me a heart attack. But the high blood pressure can.

– B16

#### 3.5.2 Ethnic Identity and Cultural Influence on Health Perceptions

Some participants expressed that they prioritised conditions that their ethnic identity made them more vulnerable to. For instance, one participant expressed this by saying, ‘I feel that diabetes is the most important thing, but they can feed off each other too, like one could make the other one worse. But I am Aboriginal, and I know diabetes kills my feet.’ – B1.

Other participants highlighted that their culturally specific diets made it difficult for them to follow advice on lifestyle modifications for a specific condition, hence making them prioritise managing the condition more with medication since lifestyle modifications were harder to implement. This theme was expressed in some interviews, as demonstrated below:

I do my best to manage my diabetes with medicine because, you know, we are Chinese people, and we like to eat rice. My GP always asked me to cut down on rice, and that is very hard for me. But with high blood pressure, it is not just about the medicine. I managed to cut down on salt and fast food, so that helps as well.

– B12

It is still under control, unlike high blood pressure, which is easy to manage. I have to make sure that I don't forget the diabetes medication because of some food restrictions that are hard to keep up with. As you know, we have a lot of different kinds of sweets, especially in the Middle East. So, I do my best to take the diabetes medication on time.

– B30

### 3.6 Condition‐Related

#### 3.6.1 Previous Experience

Participants' previous experiences with either hypertension or diabetes, particularly witnessing illness or death in close family members or friends, also influenced which condition they prioritised. For most participants, these experiences left impressions that created a fear of experiencing the same outcomes, as demonstrated in the following statements:

The diabeties medication is my first priority. And it will always be my first priority because it scares me. I have lost both parents, I lost my dad to diabetes; he had amputations and stroke and heart conditions and all of that, and my mom had it as well, and she ended up with pancreatic cancer. One of my aunties had renal failure, all those sorts of things. One of my aunties also went blind because of diabetes and those concerns. They have heightened my fear that not taking my medications will lead me to that.

– B10

I have to take my high blood pressure medicine religiously because I have seen what high blood pressure does to people. My mother had it for a long time she even had a mild stroke, and that affected her. So, I thought if I do not take the medication, that could happen to me, and I do not want that to happen. I always have my high blood pressure medication.

– B2

#### 3.6.2 Condition Control

For some participants, prioritising one condition over the other was influenced by their inability to control one of the two conditions. This was more noticeable in participants who were struggling to reach treatment goals, such as maintaining their blood pressure or blood sugar level within the normal range. One participant shared, ‘I really don't think I can get off the diabetes medication. Because my number is always up. So, based on that, it seems that there is something about it that I cannot control. I know that with diabetes, I have all kinds of problems, and I have to see my doctor every year. Check my foot every now and again. So, it is more important to me.’ – B12.

Similarly, another participant explained, ‘I would choose to take the diabetes medication over the high blood pressure one because I feel I have got my hypertension under control. And it is down to the lowest dose possible. And I would really like to get off it, but I do not think the doctors will do that. They never take you off something.’ ‐B19.

#### 3.6.3 Perceived Timeline of Consequences

Participants highlighted how the potential for immediate consequences from poor care influenced their decision to prioritise one condition over the other. A common theme was that participants prioritised diabetes as they believed that it could lead to more immediate consequences. One participant shared:

Diabetes is more important because if I do not manage it, I could be very unwell very quickly. Blood pressure itself is more longitudinal; it is good to manage, but certain problems associated with it, if it is not managed, would happen a lot later compared to the mismanagement of diabetes.

– B13

Some participant expressed the opposite view, prioritising hypertension management over diabetes for the same reason:

I know that they both have downsides, but I probably rank the blood pressure medication higher than diabetes because I think the downsides to not taking blood pressure medications occur quicker than the downsides of not taking blood sugar medications.

– B15

#### 3.6.4 Perceived Efficacy of Lifestyle Modifications

For some participants, the perceived need for medication influenced how they ranked the importance of diabetes and hypertension. A condition that could be partly managed through lifestyle modifications such as changes in diet and physical activity, was viewed as less serious. One participant explained, ‘I think keeping my blood pressure under control would be more important because with diabetes, I can do other things to help keep my sugars down. I could probably keep that under control better with lifestyle changes, but the high blood pressure is more mysterious, hence, the high blood pressure medication is more important’ – B14.

Another participant stated, ‘If I had to drop one medication, I would drop the high blood pressure one. Because with high blood pressure, I think it would also benefit me if I did exercise and lost weight.’ – B11. The perception that medication was non‐negotiable for one condition made participants prioritise that condition, while another condition that was seen as modifiable through lifestyle modification was approached with more flexibility.

## 4 Discussion

The findings of this study have shown that most participants managing coexisting diabetes and hypertension tended to prioritise one condition over the other. This condition prioritisation appears to have been shaped by patient‐related and condition‐related factors. Participants' perceptions of a condition combined with the observed effects of the condition, such as consequences of poor control and the potential risk of harm associated with the condition if left untreated significantly influenced condition prioritisation to adherence to medication. Diabetes was prioritised by most participants due to its immediate and noticeable consequences compared to the perceived slower progressing risk of cardiovascular events associated with hypertension. Additionally, our study found that condition prioritisation had a direct influence on medication adherence as people were more likely to be adherent to treatments for conditions they prioritised. Our study highlights selective adherence as a key mechanism through which participants managed multiple co‐existing conditions.

Our observations of condition prioritisation were consistent with those reported in a narrative literature review by Bratzke et al. [31], who assessed self‐management priority setting and decision making in adults with multimorbidity and highlighted that individuals with coexisting chronic conditions tended to prioritise one condition over the others. Similarly, a recent meta synthesis that assessed individuals' experiences concerning living with multimorbidity provided both qualitative and quantitative evidence to support condition prioritisation, emphasising that individuals with multiple chronic conditions frequently focused on managing the condition they perceived as most significant [10]. Although none of the studies included in the reviews focused specifically on diabetes and hypertension only, the common conditions in most of the studies included one or both conditions.

Two of the main sub‐themes identified were fear of consequences and previous experience. This emotion‐driven prioritisation was evident in participants' narratives, where the emotional weight attached to a condition or potential consequences of one condition overshadowed the other. This observation aligns with the principles of the Health Belief Model, which suggests that individuals are more likely to engage in health promoting behaviours when they perceive the consequences of an untreated condition as severe [22]. Similarly, a study by Junius‐Walker et al. [15], which assessed how older patients prioritised multiple health problems, reported that patients tended to prioritise conditions linked to physical discomfort, emotional burden, and functional distress, suggesting that negative experiences play a role in determining how patients rank conditions. Addressing subjective experiences through patient education and shared decision‐making can reduce emotionally driven condition prioritisation and improve overall medication adherence.

In addition to the impact of condition consequences highlighted above, participants' narratives revealed that the condition believed to have more immediate consequences, such as rapid deterioration, was often given priority over the one perceived to progress more slowly. In line with our results, a study that assessed risk perception and behaviour reported that as perceived risks become more immediate, individuals tended to interpret the risk as more severe [32]. Similarly, a systematic review that analysed priorities of patients with multimorbidity and of clinicians regarding treatment and health outcomes reported that patients prioritised a condition based on illness presentation, while health practitioners' prioritisation was dependent on long‐term risks [13]. To address this, interventions should educate people with coexisting diabetes and hypertension on the interconnectedness of the two coexisting conditions and the long‐term consequences of neglecting either. Results from our study also suggested that participants tended to deprioritise a condition seen as manageable with minimal intervention. Interventions should also aim to increase self‐efficacy and ability to manage the conditions themselves. This is particularly important for conditions with a bidirectional relationship like diabetes and hypertension.

Condition prioritisation led to selective adherence, with participants allocating adherence effort to treatments they perceived as more necessary. For instance, our findings showed that participants often prioritised adherence to medication for conditions they perceived as less responsive to lifestyle modification, particularly when dietary changes conflicted with established culture. The resistance to dietary modifications which reduce the perceived effectiveness of lifestyle modifications leading participants to rely more on pharmacological management for the prioritised condition highlights the importance of delivering culturally sensitive health interventions. Previous studies have reported on this, for instance, a systematic review by Vanstone et al. [33] that looked at diet modification challenges faced by people with type 2 diabetes reported that cultural and identity factors significantly impacted participants' adherence to dietary changes. To further support the reliance on pharmacological management, a systematic review by Shahin et al. [34] that assessed the impact of personal and cultural beliefs on medication adherence of patients with chronic illnesses not only reported that personal and cultural beliefs significantly affect medication adherence but went on to highlight that patients often rely on medications when lifestyle changes, such as dietary adjustments, conflict with cultural norms and practices. Neglecting culturally sensitive adaptations can reduce the effectiveness of medication adherence and condition prioritisation interventions.

Our findings highlighted that participants prioritised the condition they perceived themselves as being more vulnerable to. This also aligns with the principles of the Health Belief Model, which suggests that individuals are more likely to engage in health‐promoting behaviours when they believe that they are more susceptible to a condition [22]. Collectively, these findings support The Common‐Sense Model of Self‐Regulation, which explains how individuals develop and use their personal beliefs about a condition to manage their health [35]. Given the role perception of condition plays in influencing health behaviours, it is important to understand how it impacts patients' engagement with treatment plans and their overall approach to managing coexisting conditions. By understanding how patients view each condition, healthcare providers can help them recognise the importance of treating both conditions with the same level of attention and commitment.

While participants in our study did not report other factors that influenced condition prioritisation, previous studies have also identified factors such as treatment‐related factors and socioeconomic factors [32, 36, 37]. For instance, a study that assessed how people in their early 50 s negotiate multiple chronic illnesses and everyday life reported that prioritisation may be influenced by the need to maintain valued social roles and meet social expectations [37]. In contrast, our participants highlighted the impact of these factors on medication adherence but not condition prioritisation. These findings suggest that interventions would benefit from adopting a multifactorial approach that acknowledges the interplay of various factors.

Our results suggest that people with coexisting diabetes and hypertension may be more likely to adhere to treatment for the condition they perceive as more important. The variation in adherence scores indicated that subjective factors such as previous experience can influence medication adherence. While the pattern was not entirely consistent, in several cases, the condition with higher adherence corresponded to the one the participant emphasised in their narrative. The phenomenon of selective adherence has been reported in previous research, for instance a study by Malik et al. [38] that explored the effectiveness of a community pharmacy diabetes and hypertension programme reported different adherence rates to hypertension and diabetes medication pre and post intervention. However, this study did not indicate if participants were educated on how the two conditions influence each other and the importance of treating both conditions equally. Understanding that condition prioritisation affects medication adherence highlights the need for tailored interventions that integrate discussions on condition prioritisation, medication adherence and the overall impact on health outcomes.

This study identifies condition prioritisation as an important mechanism in shaping selective adherence. However, it is important to consider that condition prioritisation does not operate in isolation as there are other additional factors, such as side effects, regimen complexity, and treatment burden, which influence medication adherence [3, 10, 18]. Selective adherence, therefore, reflects the interaction between prioritisation processes and other factors. Future research should explore how selective adherence is shaped, considering the role of condition prioritisation and other factors influencing adherence.

The literature on impact of condition prioritisation on medication adherence is limited, and our study contributes to this area by providing qualitative insights into factors influencing condition prioritisation and its impact on medication adherence. By identifying selective adherence in the context of multimorbidity, this study builds on existing adherence models, which usually assume people follow treatment the same way for all their conditions. Existing adherence frameworks have made important contributions to understanding medication‐taking behaviours by highlighting barriers and facilitators of medication adherence [3, 19, 21]. However, most of these frameworks look at adherence from a single disease state perspective, with the few that explore multimorbidity treating adherence as relatively uniform within conditions. By not considering condition prioritisation, existing adherence frameworks overlook drivers of selective adherence, thereby reducing the effectiveness of interventions. These findings suggest that healthcare systems should strengthen adherence support for people with multiple chronic conditions by including discussions about condition prioritisation and selective adherence within integrated models of care.

Our findings collectively demonstrate that participants' medication‐taking behaviours align with their perspectives on and experiences with potential harm. These results are consistent with those of Eshete et al. [39] who reported a statistically significant association between higher illness perception and increased medication adherence in patients with diabetes. Participants' narratives suggested differences in understanding of hypertension and diabetes, which may have shaped how conditions were prioritised. Future research could explore how health literacy influences prioritisation and selective adherence in individuals with co‐existing conditions. Our study has some limitations that should be considered when interpreting these results, such as reliance on self‐reported data, which introduces the potential of recall bias and social desirability bias. All interviews were conducted in English, and eligibility required participants not to need a translator, which may have limited linguistic diversity despite the inclusion of participants from diverse ethnic backgrounds. Participants were recruited via a market research panel and received financial incentives, which may introduce selection bias and limit generalisability. Additionally, medication adherence was measured using an indirect method, which is subject to bias and does not confirm medication ingestion.

## 5 Conclusion

Most participants managing coexisting diabetes and hypertension prioritised one condition over the other based on factors such as fear of consequences and perceived condition control. Condition prioritisation amongst this group influenced their medication adherence, with participants adhering more to the medication for the condition they prioritised. To address this, future interventions should aim to address factors that influence condition prioritisation and include specific patient education on the conditions, their interconnection, as well as self‐management.

## Author Contributions

**Pauline Tendai Maniki:** conceptualisation, writing – original draft, methodology, validation, writing – review and editing, project administration, data curation, formal analysis, investigation. **Betty Bouad Chaar:** conceptualisation, supervision, writing – review and editing, validation, methodology, formal analysis. **Parisa Aslani:** conceptualisation, investigation, methodology, validation, writing – review and editing, formal analysis, supervision.

## Funding

The authors have nothing to report.

## Ethics Statement

Ethics approval was obtained from The University of Sydney Human Ethics Committee (Project number: 2023/584). All participants provided consent before taking part in the interviews.

## Conflicts of Interest

The authors declare no conflicts of interest.

## Supporting information

Supporting File 1

Supporting File 2

Supporting File 3

Supporting File 4

## Data Availability

The qualitative datasets generated and analysed during the current study are not publicly available to protect participant confidentiality. Further information may be requested from the corresponding author, subject to ethical approval.
