# Supplementary material for: A Qualitative Study to Explore the Influence of Condition Prioritisation in People With Coexisting Diabetes and Hypertension on Medication Adherence
Source: Health Expect. 2026 May 4;29(3):e70682. doi: 10.1111/hex.70682 (PMC13139724; doi:10.1111/hex.70682)
Supplement: Supplementary file 1 — Supporting File 1 [file HEX-29-e70682-s002.docx]

| **Domain 1: Research team and reﬂexivity** | | | |
| --- | --- | --- | --- |
| Personal Characteristics | | | |
| *Interviewer/facilitator* | 1 | Which author/s conducted the interview | Pauline T Maniki (PTM) |
| *Credentials* | 2 | What were the researcher’s credentials? E.g. PhD, MD | BPharm, MPharm |
| *Occupation* | 3 | What was their occupation at the time of the study? | PhD candidate and registered pharmacist intern |
| *Gender* | 4 | Was the researcher male or female? | Female |
| *Experience and training* | 5 | What experience or training did the researcher have? | PTM has undertaken training in qualitative research methodologies. |
| Relationship with participants | | | |
| *Relationship established* | 6 | Was a relationship established prior to study commencement? | A brief relationship was established at the start of the interview through introductions. The interviewer explained their role as a researcher, the study purpose and what participation would involve. No prior relationship existed between interviewer and the participants. |
| *Participant knowledge of the interviewer* | 7 | What did the participants know about the researcher? e.g. personal goals, reasons for doing the research | Participants knew the researcher’s role, affiliation and purpose of the research |
| *Interviewer characteristics* | 8 | What characteristics were reported about the interviewer/facilitator? e.g. Bias, assumptions, reasons and interests in the research topic | The researcher is a registered pharmacist intern and has an interest in the research topic and that she is completing it as part of her Ph.D in Pharmacy. |
| **Domain 2: study design** | | | |
| Theoretical framework | | | |
| *Methodological orientation and*  *Theory* | 9 | What methodological orientation was stated to underpin the study? e.g. grounded theory, discourse analysis, ethnography, phenomenology, content analysis | A phenomenological approach was used to explore participants’ lived experiences of managing coexisting diabetes and hypertension. The interview guide was guided by the Health Belief model. The study used a thematic analysis approach as described by Braun and Clarke’s framework |
| Participant selection | | | |
| *Sampling* | 10 | How were participants selected? e.g. purposive, convenience, consecutive, snowball | Purposive sampling was used. Participants were recruited through a market research company with access to a diverse panel of individuals meeting the study’s inclusion criteria |
| *Method of approach* | 11 | How were participants approached? e.g. face-to-face, telephone, mail, email | Potential participants were approached by a market research company using their existing panel database. The company contacted individuals via email or telephone, based on pre-specified eligibility criteria provided by the research team. |
| *Sample size* | 12 | How many participants were in the study? | 30 participants |
| *Non-participation* | 13 | How many people refused to participate or dropped out? Reasons? | 1  Dropped out due to unavailability |
| *Setting of data collection* | 14 | Where was the data collected? e.g. home, clinic, workplace | All interviews were conducted remotely using the Zoom video conferencing platform. Participants joined from a location of their choice |
| *Presence of non-participants* | 15 | Was anyone else present besides the participants and researchers? | Only the participants and the researchers were present in the interviews. |
| *Description of sample* | 16 | What are the important characteristics of the sample? e.g. demographic data, date | Participants who were 18 years and older and on medication for both diabetes and hypertension who resided in Australia at the time of the study. |
| Data collection | | | |
| *Interview guide* | 17 | Were questions, prompts, guides provided by the authors? Was it pilot tested? | An interview guide with prompts was developed and used during the interviews. These were extensively reviewed within the research team and piloted. The interview guide has been attached as additional material 2. |
| *Repeat interviews* | 18 | Were repeat interviews carried out? If yes, how many? | Repeat interviews were not performed and do not apply to this study. |
| *Audio/visual recording* | 19 | Did the research use audio or visual recording to collect the data? | An audio recording was used to collect the data. |
| *Field notes* | 20 | Were ﬁeld notes made during and/or after the interview or focus group? | Field notes were made right after each interview |
| *Duration* | 21 | What was the duration of the interviews or focus group? | Interviews’ duration ranged from 30 to 60 minutes. |
| *Data saturation* | 22 | Was data saturation discussed? | Data saturation was reached at interview 24 and discussed. Six more interviews were done after saturation for data confirmation. |
| *Transcripts returned* | 23 | Were transcripts returned to participants for comment and/or correction? | Transcripts were not returned to participants |
| Domain 3: analysis and ﬁndings  Data analysis | | | |
| *Number of data coders* | 24 | How many data coders coded the data? | The first two interviews were coded by two independent coders, and the rest were coded by PTM. |
| *Description of the coding tree* | 25 | Did authors provide a description of the coding tree? | A coding framework has been provided as an appendix. |
| *Derivation of themes* | 26 | Were themes identiﬁed in advance or derived from the data? | Themes were derived from the data |
| *Software* | 27 | What software, if applicable, was used to manage the data? | NVivo 14 |
| *Participant checking* | 28 | Did participants provide feedback on the ﬁndings? | No |
| Reporting | | | |
| *Quotations presented* | 29 | Were participant quotations presented to illustrate the themes / ﬁndings? Was each quotation identiﬁed? e.g. participant number | Quotations have been presented with participant codes assigned to all participants and used against quotations |
| *Data and ﬁndings consistent* | 30 | Was there consistency between the data presented and the ﬁndings? | We endeavored to report the study findings in a clear, consistent manner to accurately reflect the data that have been collected |
| *Clarity of major themes* | 31 | Were major themes clearly presented in the ﬁndings? | Yes, major themes are clearly presented |
| *Clarity of minor themes* | 32 | Is there a description of diverse cases or discussion of minor themes? | The analysis primarily reports major themes, but variations and outlier cases are described where relevant to enrich interpretation. |
