# Supplementary material for: A Qualitative Study to Explore the Influence of Condition Prioritisation in People With Coexisting Diabetes and Hypertension on Medication Adherence
Source: Health Expect. 2026 May 4;29(3):e70682. doi: 10.1111/hex.70682 (PMC13139724; doi:10.1111/hex.70682)
Supplement: Supplementary file 2 — Supporting File 2 [file HEX-29-e70682-s004.pdf]

Semi-Structured interview protocol

Exploring medication adherence and disease management in patients with coexisting High blood pressure and diabetes: A qualitative study with communities in Sydney, Australia.

Objectives

1. To understand patients' perceptions of having and managing High blood pressure and diabetes.

2. To investigate factors that impact patients' adherence to diabetes and High blood pressure medication.

3. To explore patients' medication-taking behaviour.

4. To explore strategies used by patients to optimize adherence to prescribed diabetes and High blood pressure pharmacological management.

Introduction

Hello --------- (Self-intro); **Verbal Consent** (Do you consent to be a participant in this study).

Thank you for agreeing to take part in this study. As you have read in the information sheet about the study, we are conducting a study to find out how people who have diabetes and high blood pressure take their medicines, and any issues that they may have with them. This interview will last approximately 30 - 60 minutes. I would like to remind you that we will be recording this interview, then transcribing what you have said, and then destroying the video and audio records. Are you okay with this? There is no right or wrong answer, and this interview will help us to find ways of supporting people taking medicines for diabetes and high blood pressure.

Interview Section	Questions	Prompts
**Medication history**<br>*Initial diagnosis of High blood pressure and diabetes.*<br><br>*Medication initiation*	**Diabetes**<br>➢ When were you diagnosed with diabetes?<br>➢ How did you get this diagnosis?	• Who gave the diagnosis?<br>• How long after your diagnosis did you start on medication for diabetes?<br>*Initiation gap, Reasons for delay?
	**High blood pressure**<br>➢ When were you diagnosed with High blood pressure?<br>➢ How did you get this diagnosis?	• Who gave the diagnosis?<br>• How long after your diagnosis did you start on medication for High blood pressure?<br>*Initiation gap, Reasons for delay?

**Chronic conditions perception**<br><br>*How the participant's perception of having each condition has evolved over time.*<br><br>*Patient's perception of having both conditions*	**Research has shown that people who are diagnosed with diabetes, go through a number of emotions.**<br>**Diabetes**<br>➢ Can you describe how you felt when you were first diagnosed with diabetes?<br>➢ How do you feel now about living with diabetes?<br>**High blood pressure**<br>➢ Can you describe how you felt when you were first diagnosed with High blood pressure?<br>➢ How do you feel now about living with High blood pressure?<br>➢ How do you feel about having both High blood pressure and diabetes?	• Is there anything else you would like to add or share about your experience of living with both conditions?
**Pharmacological management of conditions.**<br><br>*Patient's understanding of current medication and medication-taking behaviour.*<br><br>*Patient's perception of current treatment*	**Diabetes**<br>➢ How many medicines are you taking for your diabetes?<br>➢ How did the doctor tell you to take them?<br>➢ How is your experience of taking them?<br>**Most people forget to take their medicines or sometimes decide not to.**<br>➢ How are you with taking your diabetes medicine as prescribed by your doctor?<br>➢ How do you think your diabetes medicines have helped you?<br>➢ Do you ever experience any problems from your diabetes medication * If negative have they discussed it with their HCP?	• Have you explored alternative or complementary therapies for diabetes? If yes, what was your experience with them?<br>• What are some of the problems that you have had with the medicine?

	**High blood pressure**<br>➢ How many medicines are you taking for your High blood pressure?<br>➢ How did the doctor tell you to take them?<br>➢ How is your experience of taking them?<br>**Most people forget to take their medicines or sometimes decide not to.**<br>➢ How are you with taking your High blood pressure medicine as prescribed by your doctor?<br>➢ How do you think your High blood pressure medicines have helped you?<br>➢ Do you ever experience any problems with your High blood pressure medication? * If negative have they discussed it with their HCP?	➢ Have you explored alternative or complementary therapies for High blood pressure? If yes, what was your experience with them?<br>➢ What are some of the problems that you have had with the medicine?
**Managing both chronic conditions.**<br><br>*Patient's perception of managing both conditions.*<br><br>*If the patient manages both conditions the same way.*	➢ How do you feel about taking medicines for both high blood pressure and diabetes?<br>**Most people with more than 1 condition don't always take medication for both conditions the same way.**<br>➢ Between diabetes and high blood pressure, do you feel that one is more important than the other when it comes to managing the illness?<br>➢ If you had the choice to take medication for 1 of the 2 conditions which one would you choose?<br>➢ If you had to stop taking any of the medications you are taking which one would you stop?<br>➢ If you had to choose taking one of the medications you are taking which one would you choose?	• What impact has having the two long-term conditions had on your daily life and activities?<br>*changes in lifestyle or outlook on life, physical, emotional, or social aspects

**Medication adherence**<br><br>*Factors affecting medication adherence.*<br><br>*Strategies used to optimize medication adherence.*<br><br>*Support patient needs to improve medication adherence*	➢ What do you think motivates you to take your medicines?<br>➢ What do you do to make sure that you take all your medicines, and on time?<br><br>**Most people experience different challenges with regards to taking their medication as instructed.**<br><br>➢ What do you think gets in the way of you taking your medicines?<br>➢ What about the medicines makes it easy for you to take it?<br>➢ What about the medicines makes it hard for you take it?<br>➢ If there was a combined pill for both conditions would you consider it?<br>➢ What are some of the issues that you have with your medicines and medicine taking?<br>➢ What support do you need to help you take your medication as instructed?	• Are there symptoms that motivate you?<br>• How do you involve family members or friends in supporting your efforts to adhere to your medications?<br><br>• How do you feel when you miss a dose of your medications?<br>*what do you do the moment you realise you missed a dose?<br>• How does having the two impact your overall medicine taking for the two?
**Medication adherence determination - ARMS**	ARMS questionnaire – Appendix F	
**Demographic Information**		**In closing, I would like to know a few things about you.**<br>(Most of it is provided by market researcher i.e., gender, age, and ethnicity)<br>What is the highest education level you have completed?

Conclusion

We will then allow the participant to add any additional comments or concerns. Express gratitude for their participation and input. Remind them of the confidentiality of their responses.

End of interview.
