# Supplementary material for: A Qualitative Study to Explore the Influence of Condition Prioritisation in People With Coexisting Diabetes and Hypertension on Medication Adherence
Source: Health Expect. 2026 May 4;29(3):e70682. doi: 10.1111/hex.70682 (PMC13139724; doi:10.1111/hex.70682)
Supplement: Supplementary file 3 — Supporting File 3 [file HEX-29-e70682-s001.docx]

**Additional material 3 - Representative quotes**

| **Theme/subtheme** | **Illustrative quote** |
| --- | --- |
| **Patient -related** |  |
| Fear of consequences | “Well, I think diabetes is more important just because of the complications there are if you've neglected and stopped taking medication, which can be very dangerous.” – B21 |
|  | “Yes, my diabetes is more important to manage than my high blood pressure. Because if I don't manage my diabetes, then diabetes will contribute to my high blood pressure.” – B28 |
|  | “Diabetes is manageable, but hypertension is scary for me, I know it can cause this not-friendly disease which attacks badly. Yeah, it's worse” – B6 |
|  | “I'm thinking that the blood pressure should be treated more effectively because it seems more severe than diabetes. And not that there has been any heart conditions in my family. But just from everything you hear about high blood pressure can be the cause of lots of different things, you know.” - B5. |
| Ethnic identity and cultural influence on health perceptions | “I would say diabetes is more important for me because I hate having to watch what I eat. My family is European. They came from the Netherlands, and bread and potatoes are their life. So yeah, that's a bit challenging to let go of, and if I don’t take the medication after eating all that, my sugar levels will be too high” - B18 |
|  | “Diabetes has always been my main concern because in my family it’s just everywhere. My parents had it, my grandparents had it, so it felt almost expected for me to have it. My background already puts me at a higher risk, hence I take it more seriously than the blood pressure” – B9 |
| **Condition-related** |  |
| Previous Experience | “I thought the diabetes one was more important until the doctor increased my blood pressure dose when my blood pressure was not good. Yeah, so now I know it's more important because I know that it can cause problems if it's too high.” – B5 |
|  | “To me, diabetes is more important. I grew up with diabetes because my mum had it, and I also had gestational diabetes. I have always known about the disease, and how crippling it is, so I take it very seriously.” – B7 |
| Condition control | “Yeah, I mean, Hypertension is not bad, its just there and doesn’t change anything, and my numbers are good. I'm not complaining, but it is what it is. But diabetes can be hard work, and it can be complex. I need to always be on top of it because if anything goes wrong, it goes downhill quickly. I have to work out how much insulin I should have before I eat, you know, that sort of stuff. So it's more important ” – B23 |
|  | “The blood pressure is very good. It is excellent. Actually, I regularly have that test when I go to the doctor and I don't worry about that much now. I am a little bit blase with high blood pressure but still trying to chase diabetes. I am very cautious with diabetes because it's taking time to stabilise .” – B19 |
|  | “I believe I've got my diabetes well under control. And there have been some periods of time when I have not been able to get my medication for two days in a row, and nothing happened, but with the high blood pressure, I get headaches from missing just a day, and my numbers are all over the place. So yeah, I can't miss a dose” – B14 |
| Perceived timeline of consequences | “Diabetes is such an inconvenience because its effects are immediate, so you have to always prioritise it. The moment my blood sugar starts to get high, I start to feel sick. And this is different from high blood pressure, I do not feel anything at all. As much as it has its effects, it takes a long time of negligence to experience them” – B30 |
|  | “I know that they both have downsides, I would probably say blood pressure medication is less important because the downsides do not occur as quickly as the downsides of not taking blood sugar medications”– B11 |
|  | “I would say high blood pressure is more important because there are effects of high blood pressure I feel when my blood pressure is going up. I can feel the high blood pressure affects me more than diabetes. It does have a different effect on the body and the mind. “- B6 |
| Perceived efficacy of lifestyle modifications | “Everybody I know has high blood pressure because they are stressed or overworked. And it's something you cannot just manage without the pills. Whereas diabetes is manageable, you have to change your lifestyle, you have to change your eating habits, you have to exercise more.” – B8 |
|  | “With my blood sugar, I watch my diet, and that made a huge difference on my sugar levels, but nothing changed for my blood pressure, so I have to manage the blood pressure medication effectively since it’s the only real option.” – B27 |
