# Supplementary material for: A Qualitative Study to Explore the Influence of Condition Prioritisation in People With Coexisting Diabetes and Hypertension on Medication Adherence
Source: Health Expect. 2026 May 4;29(3):e70682. doi: 10.1111/hex.70682 (PMC13139724; doi:10.1111/hex.70682)
Supplement: Supplementary file 4 — Supporting File 4 [file HEX-29-e70682-s003.docx]

**Additional Material 4**

Table 4 Individual adherence scores and self-reported condition prioritisation.

| **Participant** | **Condition identified as more important** | **Adherence score for diabetes** | **Adherence score for hypertension** |
| --- | --- | --- | --- |
| B1 | Diabetes | 20 | 20 |
| B2 | Diabetes | 18 | 17 |
| B3 | Equal* | 12 | 12 |
| B4 | Diabetes | 13 | 15 |
| B5 | Equal | 16 | 14 |
| B6 | Equal | 20 | 19 |
| B7 | Equal | 12 | 12 |
| B8 | Equal | 13 | 13 |
| B9 | Equal | 20 | 22 |
| B10 | Diabetes | 18 | 26 |
| B11 | Equal | 13 | 16 |
| B12 | Equal | 12 | 15 |
| B13 | Diabetes | 16 | 16 |
| B14 | High blood pressure | 16 | 13 |
| B15 | Equal | 16 | 16 |
| B16 | High blood pressure | 15 | 17 |
| B17 | High blood pressure | 20 | 20 |
| B18 | Equal | 14 | 17 |
| B19 | Diabetes | 20 | 18 |
| B20 | N/S** | 12 | 12 |
| B21 | Diabetes | 13 | 16 |
| B22 | Equal | 13 | 13 |
| B23 | Diabetes | 16 | 15 |
| B24 | Equal | 19 | 19 |
| B25 | N/S | 12 | 12 |
| B26 | High blood pressure | 15 | 17 |
| B27 | Equal | 13 | 13 |
| B28 | Diabetes | 21 | 23 |
| B29 | Diabetes | 13 | 15 |
| B30 | Equal | 13 | 15 |

*Equal – Conditions are equally important

**N/S – Not specified

The ARMS-12 tool adherence score ranges from 12 (perfect adherence) to 48 (poorest adherence), with higher scores indicating lower adherence.
